# Accurate Wideband RCS Estimation from Limited Field Data Using Infinitesimal Dipole Modeling with Compressive Sensing

**DOI:** 10.3390/s25154771

**Published:** 2025-08-02

**Authors:** Jeong-Wan Lee, Ye Chan Jung, Sung-Jun Yang

**Affiliations:** Department of Electronic Engineering, Seoul National University of Science and Technology (Seoultech), Seoul 01811, Republic of Korea; leejeongwan@seoultech.ac.kr (J.-W.L.); challion@seoultech.ac.kr (Y.C.J.)

**Keywords:** infinitesimal dipole modeling, radar cross section, scattering point

## Abstract

This communication presents an accurate and computationally efficient approach for wideband radar cross-section (RCS) estimation and scattering point reconstruction using infinitesimal dipole modeling (IDM) with compressive sensing. The proposed method eliminates the need for field sampling at numerous frequency points across the wideband range through Green’s function adjustment. Additionally, compressive sensing is employed for induced current calculation to reduce both frequency and angular sampling requirements. Numerical validation demonstrates that the method achieves a 50% reduction in field sample data and an 82.3% reduction in IDM processing time while maintaining comparable accuracy through Green’s function adjustment. Furthermore, compared to approaches without compressive sensing, the method shows a 55.1% and a 75.5% reduction in error in averaged RCS for VV-pol and HH-pol, respectively. The proposed method facilitates efficient wideband RCS estimation of various targets while significantly reducing measurement complexity and computational cost.

## 1. Introduction

Electromagnetic scattering analysis is essential for radar target recognition and identification. Scattering points represent dominant reflection points on radar targets, enabling physically meaningful target characterization and radar cross-section (RCS) estimation from sampled field data [1,2,3,4,5,6,7,8,9,10,11]. Inverse synthetic aperture radar (ISAR) method have been widely used for scattering point extraction from wideband data using fine frequency steps [2,3,4,5,6]. However, achieving high accuracy requires extensive measurements with fine frequency sampling across wide frequency range and dense angular interval, increasing measurement costs. Furthermore, when ISAR-based methods rely on finite frequency sampling, accuracy suffers at the spectral boundaries, confining reliable RCS estimation to the central frequency region [6,12].

To address these limitations, compressive sensing approaches [12,13,14,15] have been introduced. These methods employ sparse recovery algorithms, with basis pursuit denoising (BPDN) being one of the most widely used l1-regularized optimization approaches for finding sparse solutions. When scattered field data and scattering point models are formulated as an under-determined problem, compressive sensing techniques can improve RCS estimation accuracy at frequency extremes [12,14,15]. However, these approaches still face practical constraints: RCS estimation remains limited to discretely sampled frequency points, and measurement costs are still significant due to the required dense frequency sampling for wideband far-field data extraction.

To address this issue, this communication presents a wideband RCS estimation method using infinitesimal dipole modeling (IDM) with compressive sensing and coarse frequency step field data. IDM is an electromagnetic source modeling technique that employs multiple infinitesimal dipoles (IDs) as point current sources to represent complex electromagnetic structures [16,17,18,19,20,21,22,23,24,25]. Among electromagnetic source reconstruction techniques, IDM has emerged as the state-of-the-art method due to its capability to reconstruct fields at arbitrary observation points without resolution limitations inherent in Fourier-based approaches, as experimented in [20]. The proposed method efficiently determines wideband ID parameters by utilizing the frequency correlation between Green’s function and the wideband scattered field. This enables efficient wideband IDM parameter determination through wave number adjustment in Green’s function, achieving RCS reconstruction at arbitrary frequency and observation points without dense sampling across the wideband range. Additionally, compressive sensing further reduces field sampling requirements for both frequency and angular measurements. This approach decreases measurement costs compared to conventional IDM methods [12,15,20,22,23] while enabling accurate scattering point reconstruction from limited field data and RCS estimation at arbitrary points. It should be noted that since the IDM formula can reflect spherical wave characteristics of near-field data without approximation, it mitigates the measurement cost challenges associated with far-field requirements for large-scale objects [26,27]. The main contributions of this work are (1) utilizing frequency correlation between Green’s function and wideband ID parameters through Green’s function adjustment, enabling efficient wideband RCS reconstruction without dense frequency sampling; and (2) employing compressive sensing with BPDN solver to reduce both frequency and angular sampling requirements while maintaining accuracy.

In the remainder of this communication, the IDM formulation for reconstructing scattering points is introduced in Section 2, and the numerical validation of the proposed wideband IDM method compared to the conventional IDM method is presented in Section 3, demonstrating the effectiveness of the proposed method. The communication concludes with Section 4.

## 2. Wideband IDM Formula with Limited Scattered Field Data

### 2.1. Dyadic Green’s Function Expression and IDM Formula

The scattered current distribution due to an incident wave can be considered an equivalent source for re-radiation. To accurately model this re-radiation process, the dyadic Green’s function formalism [28] is employed, which is widely used in electromagnetic scattering analysis such as the method of moments (MoM) [29]. The dyadic Green’s function $\bar{\bar{G}}\left(r_{\mathrm{obs}},r^{\prime };k\right)\in C^{3\times 3}$, which relates the current source to the radiated electric field, is expressed as:

(1) $$\begin{array}{c} \bar{\bar{G}}\left(r_{\mathrm{obs}},r^{\prime };k\right)=\left(\bar{\bar{I}}+\frac{1}{k^{2}}\nabla \nabla \right)\frac{e^{-jkR}}{4\pi R} \end{array}$$

where $\bar{\bar{I}}\in R^{3\times 3}$ is the unit dyadic tensor, *k* is the wavenumber, and $R=|r_{\mathrm{obs}}-r^{\prime }|$ is the distance between the observation point and current source point. This operator form of the dyadic Green’s function captures the vector nature of electromagnetic fields and accounts for both the direct radiation (through the unit dyadic $\bar{\bar{I}}$) and the gradient contributions that arise from the vector potential formulation. The expanded form of the dyadic Green’s function is explicitly expressed as:

(2) $$\begin{array}{c} \bar{\bar{G}}\left(r_{\mathrm{obs}},r^{\prime };k\right)=\frac{e^{-jkR}}{4\pi R}\left[\left(1+\frac{jkR-1}{k^{2}R^{2}}\right)\bar{\bar{I}}+\frac{3-3jkR-k^{2}R^{2}}{k^{2}R^{2}}\hat{R}\hat{R}\right] \end{array}$$

where $\hat{R}=\left(r_{\mathrm{obs}}-r^{\prime }\right)/R$ is the unit vector from the current source to the observation point. Since this formula includes both near-field terms ($1/R^{2}$ and $1/R^{3}$ dependence) and far-field terms (1/R dependence), spherical wave characteristics of both near-field and far-field are reflected without any approximation.

**Figure 1:**
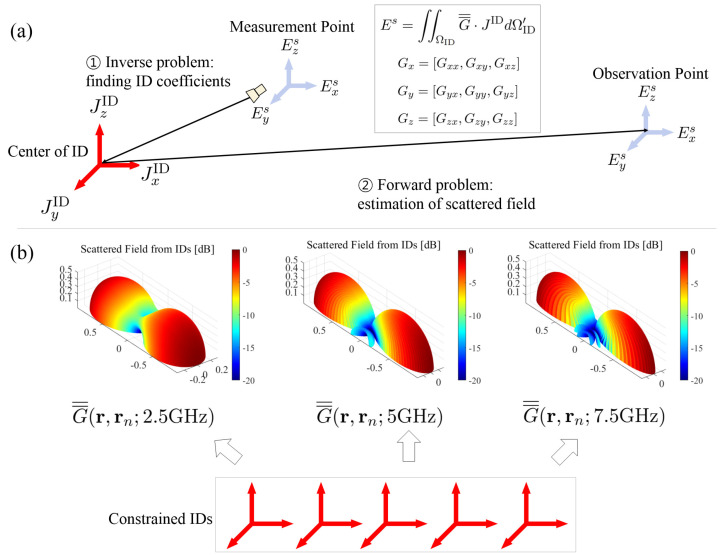
Illustration for the (**a**) IDM process and (**b**) proposed Green’s function adjustment.

Based on this (1), (2), IDM is employed to accurately identify the equivalent sources from the scattered field. As shown in Figure 1a, the IDM process is composed of finding ID complex coefficients from the scattered field (inverse problem) and estimating the scattered field from these coefficients (forward problem). IDM represents complex scattering points as collections of infinitesimal dipoles (IDs) with specific positions, orientations, and complex excitation coefficients. The IDM technique employed in this communication has been validated against measured data in [20,21], demonstrating reliable reconstruction of electromagnetic scattering characteristics. To reduce computational complexity while maintaining accuracy, a constrained IDM approach is applied [17,18,19,20,21], fixing the positions and orientations of the dipoles while optimizing only the excitation coefficients. Three orthogonal IDs (aligned with the *x*, *y*, and *z* axes) are placed at each grid point in the boundary plane surrounding the scattering object, as shown in Figure 1. This configuration ensures that arbitrary current distributions can be represented through the superposition of these elementary dipole sources.

In the IDM approach, the continuous current distribution ${\bar{J}}^{\mathrm{ID}}\left(r\right)\in C^{3\times N}$ is discretized using infinitesimal dipoles positioned at specific *N* grid points:

(3) $$\begin{array}{c} {\bar{J}}^{\mathrm{ID}}\left(r\right)=\sum_{n=1}^{N}\sum_{p=1}^{3}w_{n,p}^{\mathrm{ID}}\delta \left(r-r_{n}\right){\hat{a}}_{p} \end{array}$$

where $w_{n,p}^{\mathrm{ID}}$ is the complex coefficient of the *n*-th ID component (n=1,⋯,N) and *p*-th polarization of ID, ${\hat{a}}_{p}$ defines the dipole’s orientation (along *x*, *y*, and *z* axes), $r_{n}$ is the position vector of the *n*-th ID, and $\delta (r-r_{n})$ is the Dirac delta function that localizes each dipole source at its specific position. With the current distribution expressed in (3), the electric field at any observation point and frequency can be computed by integrating the contribution from all dipole sources. The electric field $\bar{E}\left(r_{\mathrm{obs}};k\right)\in C^{3\times 1}$ at observation point $r_{\mathrm{obs}}\in R^{3}$ and wavenumber *k* due to current sources ${\bar{J}}^{\mathrm{ID}}\left(r_{n}\right)$ distributed over the ID region $\Omega_{\mathrm{ID}}$ is given by:

(4) $$\begin{array}{c} \bar{E}\left(r_{\mathrm{obs}};k\right)=j\omega \mu {\;\int \int \;}_{\Omega_{\mathrm{ID}}}\bar{\bar{G}}\left(r_{\mathrm{obs}},r_{n};k\right)\cdot {\bar{J}}^{\mathrm{ID}}\left(r_{n}\right)d\Omega_{\mathrm{ID}} \end{array}$$

where ω is the angular frequency and μ is the permeability of free space.

### 2.2. Proposed Green’s Function Adjustment for Efficient Estimation of Wideband RCS Characteristics

For inverse scattering problems such as IDM, the scattered field is measured at discrete angular and frequency points. The measured field data can be represented as the re-radiated field from the actual induced current distribution $\bar{J}\left(r^{\prime }\right)\in V$ on the radar target. And the objective of IDM is to find the complex coefficients of IDs that re-radiate the same field as the measured field. This is formulated through the measured scattered field ${\bar{E}}^{s}\left(r_{\mathrm{meas}};k\right)\in C^{3\times 1}$ in measurement point $r_{\mathrm{meas}}\in R^{3}$ as follows:

(5) $$\begin{array}{cc} {\bar{E}}^{s}\left(r_{\mathrm{meas}};k\right) & =j\omega \mu {\;\int \int \int \;}_{V}\bar{\bar{G}}\left(r_{\mathrm{meas}},r^{\prime };k\right)\cdot \bar{J}\left(r^{\prime }\right)dV^{\prime }\\ \; & ≃j\omega \mu {\;\int \int \;}_{\Omega_{\mathrm{ID}}}\bar{\bar{G}}\left(r_{\mathrm{meas}},r_{n};k\right)\cdot {\bar{J}}^{\mathrm{ID}}\left(r_{n}\right)d\Omega_{\mathrm{ID}}^{\prime }. \end{array}$$

For wideband applications, the challenge lies in determining ID coefficients across multiple frequencies. Consider coarse frequency sampling at $k_{m}^{\prime }=k_{0}^{\prime }{+}^{\prime }2\pi m\Delta f^{\prime }$ where $m^{\prime }=0,1,\cdots ,M^{\prime }$. The wideband IDM problem at measurement points can be formulated as:

(6) $$\left[\begin{array}{c} {\bar{E}}^{s}\left(r_{\mathrm{meas}};k_{1}^{\prime }\right)\\ \vdots \\ {\bar{E}}^{s}\left(r_{\mathrm{meas}};k_{M^{\prime }}^{\prime }\right) \end{array}\right]=\sum_{n=1}^{N}{\bar{w}}_{n}^{\mathrm{ID}}\left[\begin{array}{c} \bar{\bar{G}}\left(r_{\mathrm{meas}},r_{n};k_{1}^{\prime }\right)\\ \vdots \\ \bar{\bar{G}}\left(r_{\mathrm{meas}},r_{n};k_{M^{\prime }}^{\prime }\right) \end{array}\right]$$

where ${\bar{w}}_{n}^{\mathrm{ID}}={\left[w_{n,1}^{\mathrm{ID}},w_{n,2}^{\mathrm{ID}},w_{n,3}^{\mathrm{ID}}\right]}^{T}\in C^{3\times 1}$ is the coefficient vector for the *n*-th ID. The main contribution of the proposed approach is utilizing the frequency correlation inherent in Green’s function (1), (2).

While wavenumber adjustment for predicting radiation patterns at non-sampled frequencies has been explored in [25], the approach was demonstrated over a relatively narrow bandwidth (2.8 GHz to 3.2 GHz) and relied solely on center frequency measurements, leading to reduced accuracy at band edges. The present work extends this concept to ultra-wideband applications, where the proposed Green’s function adjustment method achieves reduced frequency sampling requirements while preserving reconstruction accuracy across the entire bandwidth. Therefore, unlike conventional IDM approaches [12,15,20,22,23] that are limited to scattered field reconstruction only at measured frequency points, the proposed approach enables scattered field reconstruction at arbitrary frequency points by generating the Green’s function matrix through wavenumber adjustment, as shown in Figure 1b.

**Figure 2:**
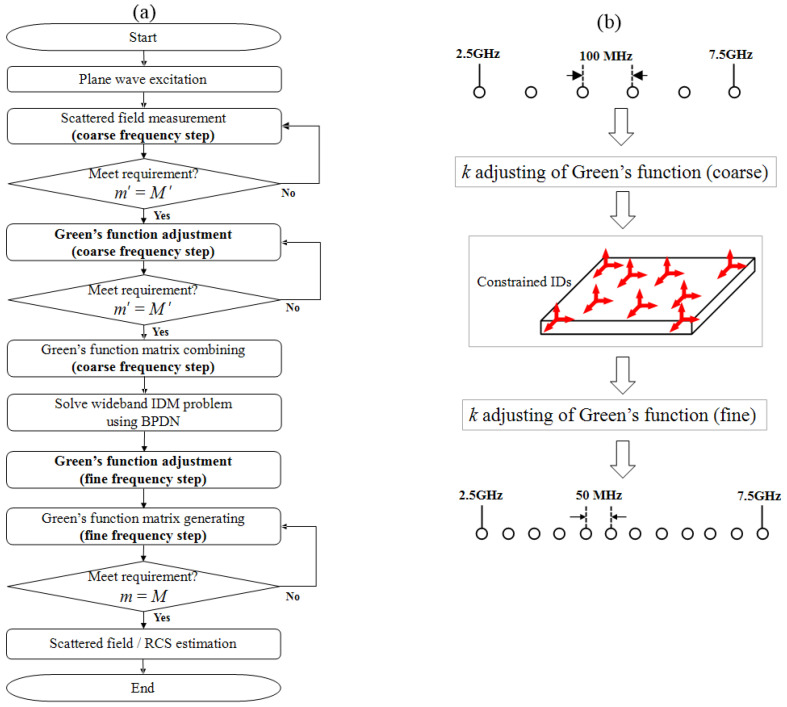
(**a**) Flowchart for proposed IDM method and (**b**) corresponding wavenumber adjusting technique of Green’s function for reducing number of required frequency sample points.

This approach assumes that within narrow frequency bands, the variation in current distribution (source) is negligible compared to the variation in Green’s function. This assumption enables us to reduce the frequency samples required to capture the wideband characteristics of RCS compared to conventional approaches, thereby significantly reducing measurement costs. Figure 2 shows the flowchart for the proposed IDM method with Green’s function adjustment technique. Once the ID coefficients ${\bar{w}}_{n}^{\mathrm{ID}}$ are determined from coarse frequency measurements, we can reconstruct the scattered field at arbitrary frequency points $k_{m}=k_{0}+2\pi \;mDeltaf$ for m=0,1,⋯,M using:

(7) $$\left[\begin{array}{c} {\bar{E}}^{s}\left(r_{\mathrm{obs}};k_{1}\right)\\ \vdots \\ {\bar{E}}^{s}\left(r_{\mathrm{obs}};k_{M}\right) \end{array}\right]=\sum_{n=1}^{N}{\bar{w}}_{n}^{\mathrm{ID}}\left[\begin{array}{c} \bar{\bar{G}}\left(r_{\mathrm{obs}},r_{n};k_{1}\right)\\ \vdots \\ \bar{\bar{G}}\left(r_{\mathrm{obs}},r_{n};k_{M}\right) \end{array}\right]$$

This enables efficient wideband scattered field ${\bar{E}}^{s}\left(r_{\mathrm{obs}};k\right)$ estimation at arbitrary observation and frequency point by:

(8) $$\begin{array}{c} {\bar{E}}^{s}\left(r_{\mathrm{obs}};k\right)=j\omega \mu {\;\int \int \;}_{\Omega_{\mathrm{ID}}}\bar{\bar{G}}\left(r_{\mathrm{obs}},r_{n};k\right)\cdot {\bar{J}}^{\mathrm{ID}}\left(r_{n}\right)d\Omega_{\mathrm{ID}}^{\prime } \end{array}$$

where the ID coefficients ${\bar{w}}_{n}^{\mathrm{ID}}$ obtained from coarse frequency measurements can be applied to reconstruct fields at any frequency *k* and observation point $r_{\mathrm{obs}}$, significantly reducing the required number of frequency samples while maintaining reconstruction accuracy. From the estimated scattered field ${\bar{E}}^{s}\left(r_{\mathrm{obs}};k\right)$, the RCS of the target can be calculated as:

(9) $$\begin{array}{c} \sigma \left(\phi ;k\right)=\lim_{r_{\mathrm{obs}}\to \infty }4\pi r_{\mathrm{obs}}^{2}\frac{|{\bar{E}}^{s}\left(r_{\mathrm{obs}};k\right){|}^{2}}{|{\bar{E}}^{i}\left(r_{\mathrm{obs}};k\right){|}^{2}} \end{array}$$

where $E^{i}\left(r_{\mathrm{obs}};k\right)$ is the incident field amplitude and ϕ denote the observation angles in spherical coordinates.

### 2.3. Wideband IDM Formula with Green’s Function Adjustment and Compressive Sensing

A major challenge in IDM is determining the optimal complex coefficients $w_{n,p}^{\mathrm{ID}}$ from limited scattered field data. Various methods using LSQR solvers have been proposed [17,18,19,20,21,22,23,24,25] providing fast and intuitive solutions. However, since the present wideband IDM problem is an under-determined problem where the number of IDs to be reconstructed exceeds the number of scattered fields, conventional approaches face limitations. In practical scattering scenarios, the induced currents exhibit sparse characteristics as the dominant scattering intensity is concentrated at and around the physical location of the target [12,15]. This sparsity property makes the sparse recovery process in compressive sensing particularly well-suited for finding solutions with limited measurement data [30]. To find the optimal complex coefficients, this communication formulates the wideband IDM problem as a BPDN problem, widely applied in compressive sensing applications for sparse signal recovery of an under-determined system. In this study, the BPDN problem is solved using the fast iterative shrinkage-thresholding algorithm (FISTA), one of the IST-based algorithms [31], which provides efficient convergence for $\ell_{1}$-regularized problems.

To represent the wideband IDM problem as a BPDN problem with regularization of the solution vector, it is formulated as:

(10) $$\min_{W^{\mathrm{ID}}}\frac{1}{2}∥GW^{\mathrm{ID}}{-E∥}_{2}^{2}+\lambda {∥W^{\mathrm{ID}}∥}_{1}$$

where E=$[{\bar{E}}^{s}\left(r_{\mathrm{meas}};f_{1}\right)$,⋯,${\bar{E}}^{s}\left(r_{\mathrm{meas}};f_{M}\right){]}^{T}$ is the sampled electric field vector, the system matrix composed of Green’s function is G=$[\bar{\bar{G}}\left(r_{\mathrm{meas}},r_{n};f_{1}\right),$⋯,$\bar{\bar{G}}\left(r_{\mathrm{meas}},r_{n};f_{M}\right){]}^{T}$ and $W^{\mathrm{ID}}={\left[w_{1,p}^{\mathrm{ID}},\cdots ,w_{n,p}^{\mathrm{ID}}\right]}^{T}$ represents the coefficients of the ID model. The regularization parameter λ is set based on the noise level of the measured data. The sparse recovery process with l1-regularization addresses the ill-posed nature of the under-determined system by promoting sparse solutions. The l1-norm penalty term $\lambda ||W^{\mathrm{ID}}{\left|\right|}_{1}$ encourages sparsity in the recovered ID coefficients, effectively selecting only the most significant scattering locations that correspond to physical scattering points on the target [30,32]. Therefore, the ill-posed inverse scattering problem is efficiently solved by a BPDN solver such as FISTA.

**Figure 3:**
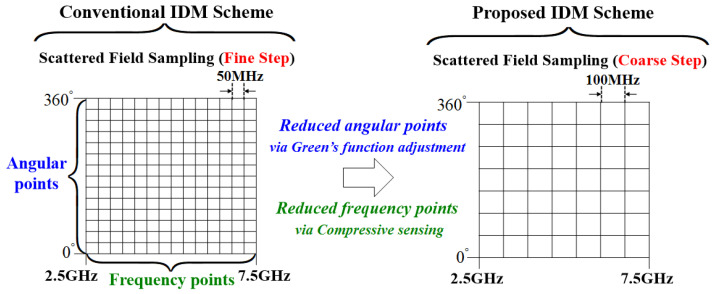
Illustration for comparison of required scattered field data between the conventional method and the proposed method for the wideband IDM problem.

The matrix size used to find the solution in (10) is proportional to *M*. Therefore, the process of finding a solution for (10) using compressive sensing with fine frequency step wideband field data takes considerable time. Furthermore, fine frequency steps increase measurement costs proportionally to *M*, and RCS estimation is limited to sampled frequency points. In contrast, the proposed method utilizes frequency correlation between Green’s function and wideband IDM parameters by adjusting the wavenumber in (1), (2), thereby enabling RCS estimation at arbitrary frequency points without dense sampling across the wideband range. Therefore, it reduces the number of frequency points by allowing coarse frequency steps in required scattered field sampling for IDM, decreasing the number of wideband measurements compared conventional IDM scheme, as shown in Figure 3. Additionally, by solving the wideband IDM problem through compressive sensing techniques such as BPDN, the required number of samples can be reduced in both frequency and angular domains. The numerical validation of the proposed wideband IDM scheme is presented in the following section.

## 3. Numerical Results for Proposed Method

**Figure 4:**
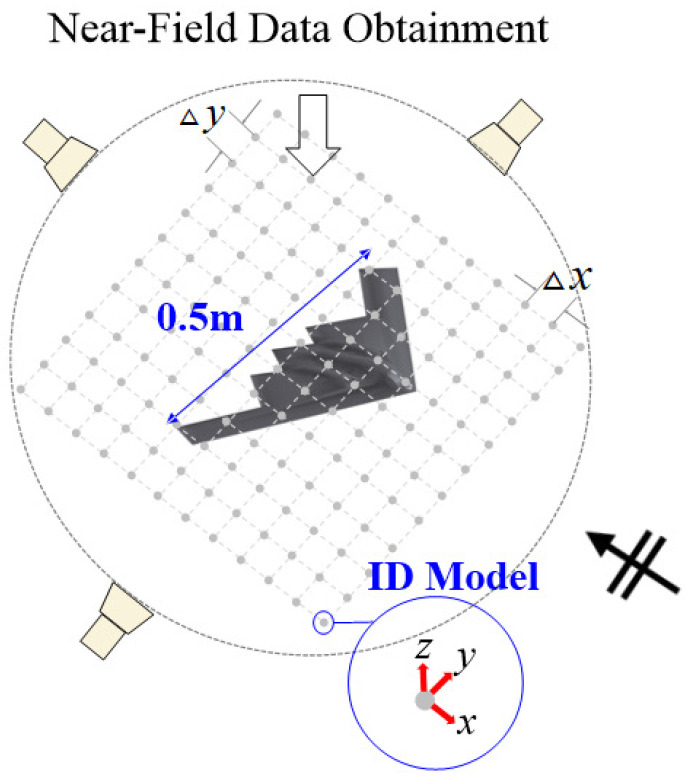
Illustration for the B-2 scaled model and locations of constrained IDs used in numerical experiment.

To evaluate the performance of the proposed wideband IDM scheme, numerical validation was performed using a scaled B-2 aircraft model with a maximum length of 0.5 m as the target, as shown in Figure 4.

The scattered field data was obtained through MoM simulation using FEKO software in a bistatic scenario, with measurements taken at a distance of 5 m from the target center. Plane wave excitation was configured with incidence direction at $\theta =90^{\circ }$, $\phi =0^{\circ }$, and full-wave simulations were conducted for both VV-polarization and HH-polarization scenarios. For the VV-polarization and HH-polarization scenarios, both incident and scattered fields were vertically polarized in the VV case and horizontally polarized in the HH case. The frequency band was set from 2.5 GHz to 7.5 GHz, with field data acquired at 50 MHz steps for the ‘Fine’ frequency step data, and 100 MHz steps for the ‘Coarse’ frequency step data. To compare the performance of the proposed method with reduced measurements, numerical validation was conducted with four different methods based on the solver type of IDM problem and the frequency sampling step: (1) BPDN-Coarse (proposed), (2) BPDN-Fine, (3) LSQR-Coarse, and (4) LSQR-Fine. For example, ‘BPDN-Coarse’ represents results for the scattering point reconstruction using BPDN-based IDM with a relatively coarse frequency resolution. For the BPDN solver, the regularization parameter λ was set to 1. The scattered field was sampled at fixed elevation angle $\theta =90^{\circ }$ while varying azimuth angle from $\phi =0^{\circ }$ to $\phi =360^{\circ }$ with $6^{\circ }$ intervals. The BPDN-based IDM approach for under-determined systems, as proposed in [12,15], represents the current state-of-the-art for wideband RCS estimation with limited field data. Note that ‘Fine’ and ‘Coarse’ methods have identical angular sampling points, but ‘Fine’ has twice as many frequency points as ‘Coarse’ due to the difference in frequency step. The conventional method, denoted as ‘BPDN-Fine’, serves as the benchmark against which we evaluate our proposed approach. For constrained IDM implementation, a square planar grid in the *x*-*y* plane spanning from −0.45 m to 0.45 m in both dimensions was used to capture the scattering characteristics of the 0.5 m target. The spacing between adjacent IDs was set to Δx=Δy=0.0138 m.

**Figure 5:**
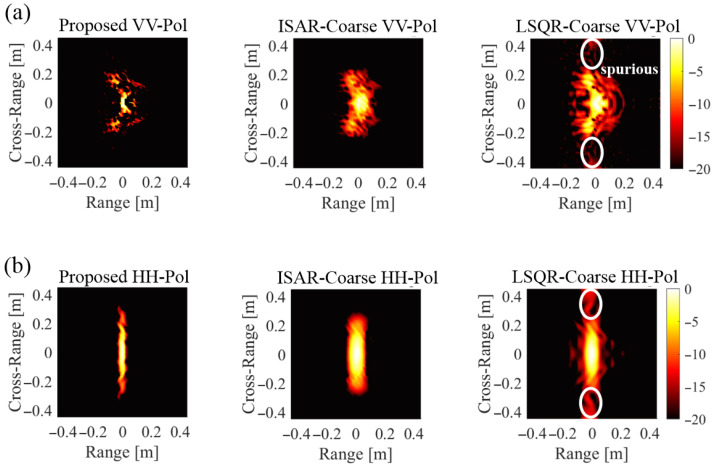
Reconstructed scattering centers for (**a**) VV-polarization, (**b**) HH-polarization.

Figure 5 compares the scattering points reconstruction results obtained from coarse frequency step data using different methods. Both VV-pol (Figure 5a) and HH-pol (Figure 5b) scenarios were analyzed. The LSQR solver produces spurious scattering points outside the 0.5 m diameter of the target. These spurious points are absent in both the ISAR [6] and BPDN results. This is because ISAR and BPDN reflect the actual locations where scattering occurs on the target, which can improve the accuracy of target identification [1,2,3,4,5]. The reconstructed scattering centers exhibit distinct spatial distributions for VV-pol and HH-pol cases, reflecting the polarization-dependent scattering mechanisms of the B-2 aircraft model. The BPDN-based reconstruction successfully identifies primary scattering regions without the spurious artifacts observed in LSQR results. The spatial localization achieved by the proposed BPDN-Coarse method demonstrates its effectiveness in preserving physical scattering characteristics.

**Figure 6:**
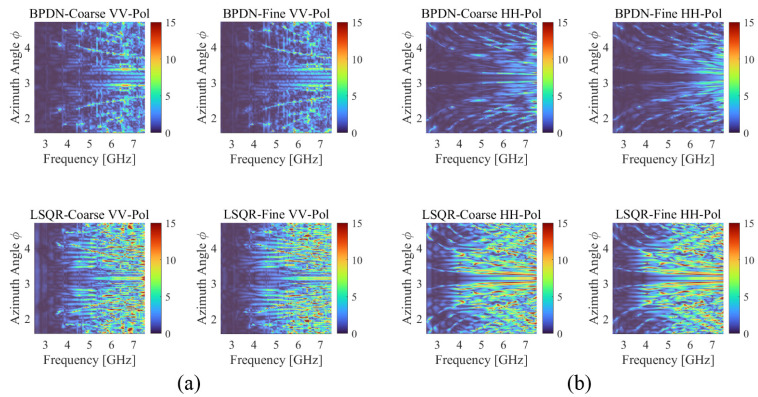
RCS error for different methods according to IDM solver type and frequency sample step. The logarithmic RCS error compared to MoM results is presented for (**a**) VV-polarization and (**b**) HH-polarization, respectively.

**Figure 7:**
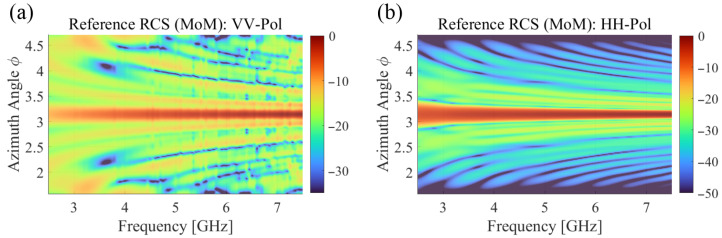
Normalized reference bistatic RCS values obtained by MoM simulations for (**a**) VV-polarization and (**b**) HH-polarization, respectively. Plane wave excitation was configured with incident direction at $\theta =90^{\circ }$, $\phi =0^{\circ }$.

Figure 6 presents logarithmic error comparisons of RCS estimation against MoM results for four different cases. The reference RCS obtained by using MoM simulations is presented in Figure 7, showing complex frequency and angular dependencies of the B-2 aircraft’s electromagnetic signature. These reference patterns serve as the ground truth for evaluating the reconstruction accuracy of different IDM methods. For both VV-pol (Figure 6a) and HH-pol (Figure 6b) scenarios, the BPDN solver consistently demonstrates lower error levels compared to the LSQR-Fine solver. As shown in Figure 7, the RCS variations along the wideband frequency range are particularly noticeable at lower frequencies (around 2.5 GHz), although the estimated RCS results in Figure 6 generally represent low errors. This implies that the proposed IDM-based RCS estimation method effectively reflects the variation in the Green’s function caused by rapid changes in electrical length at low frequencies. Notably, the ‘Coarse’ frequency step data (using half the data points of ‘Fine’ step) achieves nearly equivalent error levels to the ‘Fine’ step data for both solvers, demonstrating the accuracy of the proposed method with reduced measurements for wideband frequency points. The frequency-dependent behavior of the RCS reconstruction error reveals important insights into the proposed method’s performance characteristics.

**Figure 8:**
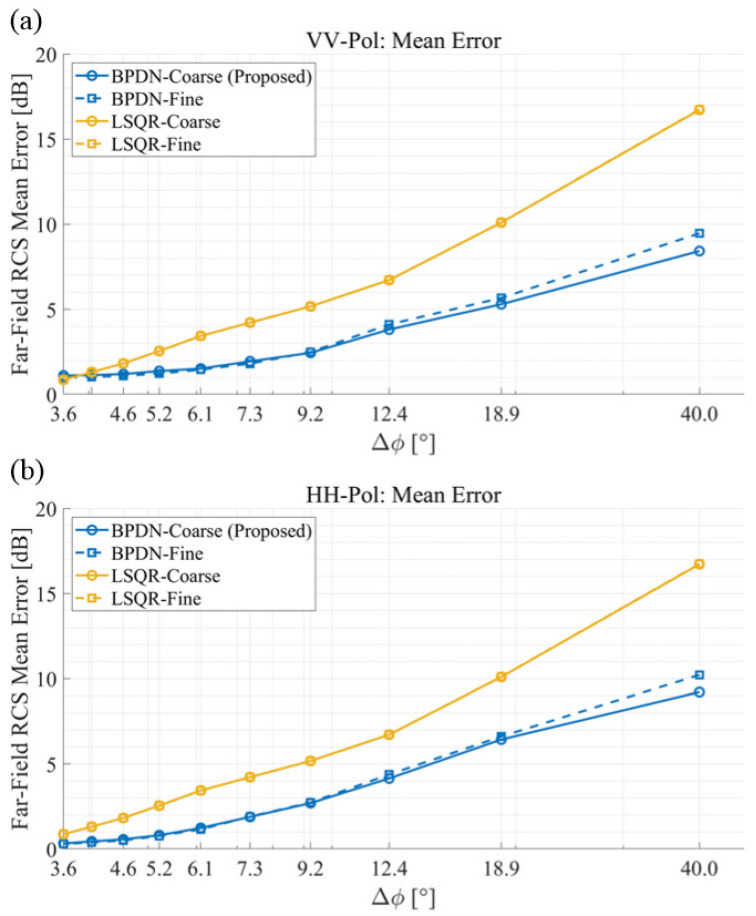
Average error of RCS according to the azimuth angular sampling interval for scattered field data. The logarithmic RCS error compared to MoM results is presented for (**a**) VV-polarization and (**b**) HH-polarization, respectively.

Figure 8 shows the average error of RCS according to azimuth angular sampling interval for scattered field data, corresponding to data reduction percentages of up to 91% ($\Delta \phi =40^{\circ }$), for the four different methods. To quantitatively evaluate and compare the errors in RCS estimation between different methods, the mean error is calculated as follows:

(11) $$\begin{array}{c} \mathrm{Mean}\;\mathrm{Error}=\frac{1}{MN_{\phi }}\sum_{j=1}^{N_{\phi }}\sum_{m=1}^{M}\left(20\log_{10}\left|\sigma^{\mathrm{MoM}}\left(\phi_{j};k_{m}\right)\right|-20\log_{10}\left|\sigma^{\mathrm{IDM}}\left(\phi_{j};k_{m}\right)\right|\right) \end{array}$$

where $\sigma^{\mathrm{MoM}}$ and $\sigma^{\mathrm{IDM}}$ denote the RCS values obtained from the MoM (reference) and IDM reconstruction, respectively, $N_{\phi }$ is the number of angular observation points, and $\phi_{j}$ represents the *j*-th azimuth angle. Larger angular sampling intervals reveal pronounced differences between BPDN and LSQR reconstruction capabilities. The angular sampling results demonstrate that the proposed BPDN-Coarse method achieves comparable accuracy with up to 91% data reduction ($\Delta \phi =40^{\circ }$, using only 9 angular samples instead of 100), while maintaining error levels below 10 dB for both polarizations. However, the results of the LSQR solver (‘LSQR-Fine’, ‘LSQR-Coarse’ in Figure 8) exhibit significantly degraded performance. This superior performance of BPDN is particularly valuable for practical measurement scenarios where reducing the number of angular samples directly translates to decreased measurement time and cost. The error curves’ slopes indicate that BPDN-based methods can achieve equivalent accuracy to LSQR methods while using approximately 50% fewer angular samples, validating the effectiveness of compressive sensing for under-sampled measurement configurations. Importantly, methods using ‘Coarse’ frequency step data achieve comparable error levels to those using ‘Fine’ step data across all angular sampling rates, demonstrating that the proposed approach enables simultaneous reduction in both frequency and angular sampling requirements without compromising reconstruction accuracy.

**Figure 9:**
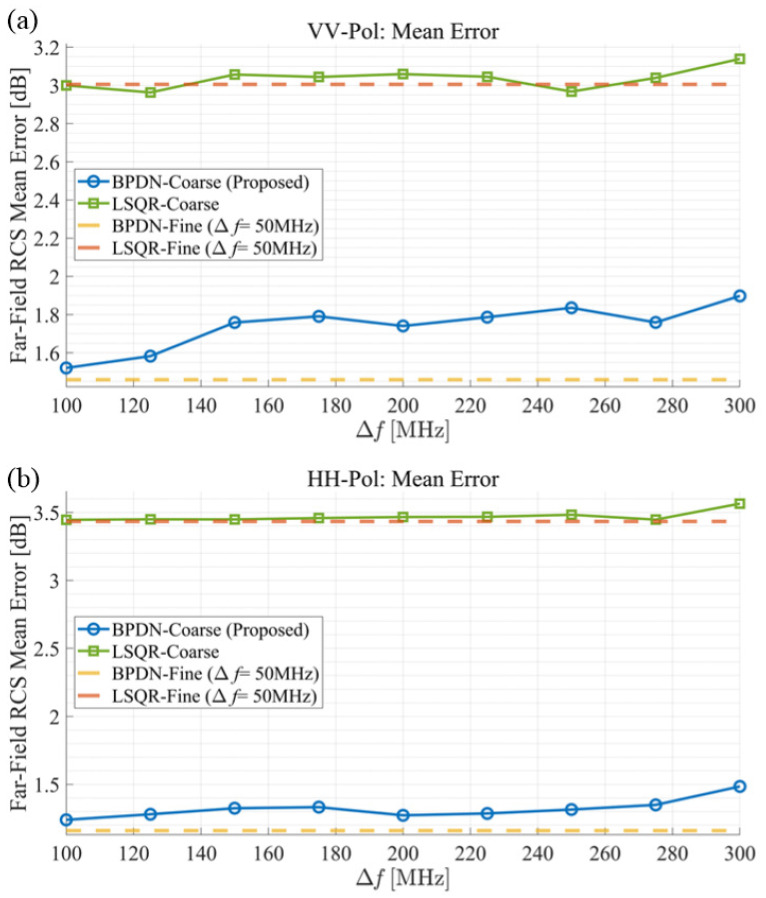
Average error of RCS according to frequency sampling steps for scattered field data. The logarithmic RCS error compared to MoM results is presented for (**a**) VV-polarization and (**b**) HH-polarization, respectively.

Figure 9 evaluates the accuracy of the proposed method across different frequency step sizes, corresponding to data reduction percentages from 50% (Δf=100 MHz ) up to 83.3% (Δf=300 MHz). When compared to the error levels of fine frequency step cases (‘BPDN-Fine’, ‘LSQR-Fine’), both VV-pol and HH-pol scenarios show only slight increases in RCS estimation error as frequency step size increases. The ‘BPDN-Coarse’ method (proposed) exhibits a nearly flat error response, indicating high tolerance to coarse frequency sampling. This behavior can be attributed to the Green’s function adjustment technique, which effectively determines wideband ID parameters by utilizing frequency correlation between Green’s function and wideband scattered field. Using the BPDN solver, the error remains below the LSQR solver error level even when the frequency step reaches six times that of the fine step (using just 16.7% of the data), with approximately 1 dB margin for VV-pol and 2 dB margin for HH-pol. This demonstrates that the proposed method can achieve RCS estimation with comparable accuracy to conventional methods while reducing measurement costs—the proposed approach allows frequency steps up to 200 MHz (reducing data acquisition by 75%) while maintaining RCS estimation errors below the levels achieved by conventional fine-step LSQR methods.

Table 1 compares the estimated RCS error and calculation time required for wideband IDM between the different methods. All four different IDM methods use identical angular sampling points with azimuth angle from $\phi =0^{\circ }$ to $\phi =360^{\circ }$ at $6^{\circ }$ intervals. The proposed method (‘BPDN-Coarse’) achieves comparable accuracy to ‘Fine’ step IDM while reducing computation time by 82.3% of the ’Fine’ step approach for both VV-pol and HH-pol scenarios. The comparison between our proposed method and BPDN-Fine effectively evaluates our approach against the state-of-the-art in source reconstruction-based wideband RCS estimation. All computations were performed using a single CPU (13th Gen Intel(R) Core(TM) i7-13700). Similar efficiency improvements are observed when using the LSQR solver, with lower overall accuracy. When comparing conventional LSQR-based results with BPDN-based results, the proposed method achieves 54.5% (VV-pol) and 75.6% (HH-pol) reduction in RCS mean error.

## 4. Conclusions

This communication demonstrated an accurate method for estimating wideband RCS through IDM with reduced measurement complexity and cost. By utilizing frequency correlation between Green’s function and wideband IDM parameters, the proposed IDM approach with coarse frequency sampling (100 MHz steps) achieved comparable accuracy to fine sampling (50 MHz steps) results with reduced processing time for IDM. Additionally, we demonstrated that the IDM approach using compressive sensing enables accurate RCS reconstruction with reduced angular samples (50%) while reducing RCS reconstruction error compared to the LSQR-based IDM approach. These advancements reduce both measurement costs and computational resources for electromagnetic characterization of complex targets, and spurious scattering points have been eliminated. Future work will focus on extending the proposed method to more challenging inverse problems, including monostatic RCS reconstruction where transmitter and receiver are co-located, and amplitude-only measurements where phase information is unavailable.

## Figures and Tables

**Table 1:** Comparison of estimated RCS error and IDM processing time for different IDM methods.

	RCS Mean Error [dB]		Processing Time
	**VV-Pol**	**HH-Pol**	**for IDM [s]**
Proposed	1.31 *	0.85 *	9.15 **
BPDN-Fine	1.22	0.88	51.50
LSQR-Coarse	2.88	3.48	0.54
LSQR-Fine	2.92	3.47	2.77

* 54.5% (VV) and 75.6% (HH) error reduction by compressive sensing. ** 82.3% time reduction by Green’s function adjusting.

## Data Availability

Data are contained within the article.
